# Effectiveness and safety of non-pharmacological therapies for the treatment of inflammatory bowel disease: a network meta-analysis

**DOI:** 10.3389/fmed.2025.1593483

**Published:** 2025-06-30

**Authors:** Jing Jia, Yun-bo Wu, Si-wei Liu, Wei-jing Chen, Ru-liu Li, Yun-long Bai, Ling Hu

**Affiliations:** ^1^Science and Technology Innovation Center, Institute of Gastroenterology, Guangzhou University of Chinese Medicine, Guangzhou, Guangdong, China; ^2^School of Chinese Materia Medica, Guangzhou University of Chinese Medicine, Guangzhou, Guangdong, China

**Keywords:** inflammatory bowel disease, non-pharmacological therapies, network meta-analysis, effectiveness, safety

## Abstract

**Background:**

Inflammatory bowel disease (IBD), encompassing both Crohn’s disease (CD) and ulcerative colitis (UC), is a chronic, inflammatory, and immune-mediated disorder of the gastrointestinal tract. If left inadequately treated, IBD can lead to disease progression, resulting in severe long-term complications, including irreversible structural damage to the intestinal tissues. While clinical symptoms are traditionally used to assess treatment efficacy, they do not always align with the underlying mucosal inflammation, particularly in CD. This limitation underscores the importance of exploring alternative treatment strategies. To address this gap, the present study evaluates the effectiveness of non-pharmacological treatments (NPTs) for IBD through a network meta-analysis (NMA), providing a thorough assessment of the available evidence.

**Methods:**

We systematically reviewed randomized controlled trials (RCTs) from the following databases: PubMed, Embase, Springer, Cochrane Controlled Register of Trials (CENTRAL), and Web of Science, comparing various NPTs for IBD, including Cognitive Behavioral Therapy (CBT), diet interventions (DI), fecal microbiota transplantation (FMT), physical training (PT), and acupuncture and moxibustion (APMX). Outcomes assessed included clinical remission, disease activity, quality of life (QOL), serum biomarkers (fecal calprotectin [FC] and C-reactive protein [CRP]), and adverse effects. The quality assessment was assessed by Cochrane Handbook and GRADEpro software. The risk ratio (RR) was calculated for dichotomous outcomes while standardized mean difference (SMD) was used for continuous variables with 95% credible intervals (CI). Funnel plot was performed to evaluate publication bias. Surface under the cumulative ranking curve (SUCRA) was conducted to rank the included interventions. Data were analyzed with STATA 15.0 and Review Manager 5.3.

**Results:**

A total of 62 eligible RCTs were identified in this NMA. The results showed that standard medical therapy (SMT) exhibited the highest probability in inducing clinical remission, as expected. Among non-pharmacological interventions, APMX, a traditional Chinese medicine involving acupuncture and moxibustion, showed promising results in both animal models and clinical trials, reducing serum TNF-α levels and improving intestinal health. DI was most effective in maintaining clinical remission and reducing serum FC levels. FMT emerged as the most effective treatment for reducing serum CRP levels and ranked second in terms of clinical remission induction.

**Conclusion:**

APMX, DI, and FMT represent promising non-pharmacological options for managing IBD. APMX was the most effective for clinical remission and symptom relief, while DI was best for maintaining remission, and FMT showed promise in reducing inflammation. Further high-quality clinical trials are needed to strengthen the evidence and guide clinical practice in IBD management.

**Systematic review registration:**

https://www.crd.york.ac.uk/PROSPERO/view/CRD42024596233, CRD42024596233.

## Introduction

Inflammatory bowel disease (IBD), encompassing both ulcerative colitis (UC) and Crohn’s disease (CD), refers to a group of chronic, inflammatory disorders of the gastrointestinal tract. The global prevalence of IBD has been steadily rising, with approximately 6.8 million individuals affected worldwide. This growing burden incurs substantial healthcare costs, amounting to billions annually, placing significant strain on healthcare systems globally (1). While the exact etiology of IBD remains unknown, it is believed to result from a combination of genetic predisposition, environmental factors, and gut microbiota dysbiosis (2, 3). Dysbiosis of the gut microbiota can activate the innate immune system, leading to the excessive secretion of pro-inflammatory cytokines (such as TNF-α, IL-6), which in turn drives chronic inflammatory cascade reactions in animal models (4). In addition, a recent prospective cohort study conducted on patients with ulcerative colitis (UC) found that patients with higher stress reactivity exhibited significant differences in their gut microbiota and metabolite profiles. These microbial and metabolic biomarkers were able to effectively predict the risk of disease relapse within the next 6 to 24 months, suggesting that the gut-brain-microbiota interactions play a crucial role in stress-related UC activity (5). Meanwhile, dysfunction of the gut-brain axis may explain the high prevalence of psychological comorbidities such as anxiety and depression in IBD patients (approximately 1/3 of patients with anxious, and 1/4 with depressed) (6), Psychological stress can further exacerbate intestinal inflammation through the vagus nerve-immune pathway, creating a vicious cycle (7–10). Despite the widespread use of traditional IBD treatment regimens in clinical practice, they all have corresponding limitations. 5-aminosalicylic acid (5-ASA) drugs have limited efficacy in Crohn’s disease and may cause side effects such as headaches and nausea, with long-term use potentially leading to kidney damage (11). Corticosteroids, while effective in inducing inflammation remission, are ineffective for maintaining long-term remission (12), and may cause adverse reactions such as bone loss, hyperglycemia, and edema (13). Immunosuppressants have a slow onset of action and may cause side effects such as leukopenia and liver damage, with long-term use potentially increasing the risk of cancer (14). Biologics, while highly effective in severe cases, carry a risk of serious infections (15) and come with high treatment costs. These limitations highlight the need for developing safer and more effective therapeutic options. Furthermore, traditional treatment plans pay insufficient attention to the mental health of IBD patients, while comorbidities such as anxiety and depression can increase the risk of disease recurrence by 1.6 times and the hospitalization rate by 42% (16). With the increasing attention to the impact of IBD on mental health, non-pharmacological therapies (NPTs) are gradually gaining importance, becoming a crucial supplement to the comprehensive management of IBD.

Non-pharmacological therapies (NPTs), Such as psychological interventions (including cognitive behavioral therapy [CBT], mindfulness-based therapy, acceptance and commitment therapy [ACT], etc.), diet interventions (DI), fecal microbiota transplantation (FMT), physical training (PT), and acupuncture and moxibustion (APMX), have been proposed as adjunctive treatments for IBD (17–21).

Systematic reviews have assessed the efficacy of these interventions. For example, a meta-analysis by Seaton et al. (22) found that emotional interventions could effectively improve inflammation markers (such as fecal calprotectin and C-reactive protein), with therapies like CBT showing better efficacy than physical exercise. A study by El et al. (23) demonstrated that fecal microbiota transplantation (FMT) provides significant clinical and endoscopic benefits in the short-term treatment of active ulcerative colitis (UC), with good safety. The latest systematic review by Yang et al. (24) also suggested that acupuncture may have potential anti-inflammatory effects (24). In an updated 2023 systematic review by Limketkai et al. (25), it was pointed out that despite the widespread attention on dietary interventions, the existing evidence quality remains low and is characterized by high uncertainty. Additionally, earlier Cochrane reviews explored the roles of dietary interventions (18) and fecal microbiota transplantation (26) in inducing and maintaining IBD remission, but their scope was limited to single intervention types and did not compare different non-pharmacological therapies across studies.

The assessment of IBD involves multiple levels of indicators. In addition to traditional clinical symptom scores, recent clinical research has placed greater emphasis on the combination of subjective and objective outcome measures. Previous studies have shown that there is often an inconsistency between the subjective symptoms reported by IBD patients and the objective degree of mucosal inflammation, particularly in patients with Crohn’s disease (CD) (27, 28). Therefore, a single clinical symptom score often cannot comprehensively reflect the disease activity. In recent years, biomarkers (such as C-reactive protein, fecal calprotectin), quality of life scales (such as IBDQ, SIBDQ), and composite scoring tools have been widely used to assist in evaluation. Based on this, this study incorporates multiple outcome indicators, including clinical remission, disease activity, quality of life, biomarkers, and adverse reactions, in order to provide a more comprehensive evaluation of the effects of non-pharmacological interventions.

For this purpose, this study aims to conduct a network meta-analysis to evaluate the efficacy and safety of five non-pharmacological interventions: CBT, DI, FMT, PT, and APMX in treating IBD. By examining a range of therapeutic options, this review seeks to provide a comprehensive overview of non-pharmacological interventions that may complement conventional treatments and improve the overall management of IBD. This study represents the first network meta-analysis comparing the efficacy of multiple non-pharmacological interventions, aiming to systematically elucidate their relative advantages, address existing evidence deficiencies in this field, and offer evidence-based guidance for clinical decision-making regarding therapeutic selection as well as future research prioritization.

## Materials and methods

This study was conducted in accordance with the Cochrane criteria, the Preferred Reporting Items for Systematic Reviews and Meta-Analyses (PRISMA) statement (29) and relevant meta-analysis guidance. The protocol has been registered with PROSPERO under the registration number CRD42024596233.

### Search strategy and study selection

A comprehensive literature search was conducted from inception through 6 November 2024, in the following databases: PubMed, Embase, Springer, Cochrane Controlled Register of Trials (CENTRAL), and Web of Science (Supplementary File 1). There were no restrictions on language or publication date. Randomized controlled trials (RCTs) meeting the PICO (Population, Intervention, Comparison, Outcome) methodology were eligible for inclusion: (1) Participants: Individuals with a confirmed diagnosis of IBD; (2) Interventions: Any non-pharmacological therapy for IBD treatment, including Cognitive Behavioral Therapy (CBT), dietary interventions (DI), fecal microbiota transplantation (FMT), physical training (PT), and acupuncture and moxibustion (APMX), the combination therapies were also included; (3) Comparisons: Comparison with usual conventional treatments, placebos, or other non-pharmacological interventions. Any study in which there is a clear difference in treatment methods between the intervention and control groups may be included for comparison. In some studies, “usual treatment” was defined as standard medical therapy (SMT), which refers to conventional pharmacological regimens for IBD (e.g., mesalazine, corticosteroids, or biologics). SMT was treated solely as a comparator and not as a non-pharmacological intervention in this analysis; (4) Outcomes: Clinical remission, disease activity, gastrointestinal symptoms, inflammation biomarkers (C-reactive protein [CRP], fecal calprotectin [FC]), quality of life (QOL), and adverse effects. Studies were excluded based on the following criteria: meeting abstracts (Since these typically do not include complete methods and results data, making it difficult to extract data and assess bias.); incomplete or imprecise data; ambiguous treatment protocols; unavailable full texts; cross-sectional studies; or reviews. Studies with a Jadad score of ≤ 3 were also excluded.

### Data extraction

Two authors independently performed data extraction from the included studies. Any discrepancies were resolved by discussion between the two independent investigators. Adjudication was performed as needed by a third author (MG). Extracted data included the study design, population characteristics, intervention, comparator, duration of interventions and follow-up, outcomes, timing, setting, the method of handling missing data, funding source, and potential conflicts of interest. In cases where data is incomplete or information is unclear in the literature, we proactively contacted the corresponding author to obtain Supplementary Information.

### Quality evaluation

Risk of bias for RCTs was independently assessed by two authors using the Cochrane Risk of Bias tool (30). The overall certainty of evidence was independently assessed by two authors for each stratified outcome using the GRADE methodology (31). Inconsistencies were resolved by a third author. Risk of bias was evaluated based on standard definitions used in Cochrane systematic reviews, with domains including random sequence generation, allocation concealment, blinding of participants and personnel, blinding of outcome assessment, completeness of outcome data, and selective reporting. Each domain was classified as “low risk,” “unclear risk,” or “high risk.” Heterogeneity was initially assessed qualitatively by considering differences in study populations (e.g., age, sex, race), research settings, methods of dietary interventions, intervention durations, and definitions or thresholds for remission. For studies exhibiting qualitative homogeneity, statistical heterogeneity was assessed using the Chi^2^ test, with a *P*-value < 0.10 indicating statistically significant heterogeneity.

### Data synthesis and statistical analysis

Evidence of direct and indirect multiple-intervention comparisons is obtained by network meta-analysis, and performing this analysis with the Bayesian framework can improve the accuracy of the results. For binary outcomes, we calculated the risk ratio (RR) with corresponding 95% confidence interval (CI). For continuous outcomes, we calculated the mean difference (MD) and corresponding 95% CI. The random effects model will be utilized for combining data if there is statistically significant variation across studies, or the fixed effects model will be chosen if there was no statistically significant heterogeneity across the studies. Given the potential clinical or methodological heterogeneity in population characteristics, intervention methods, and study designs, among other aspects, across the included studies, this study uniformly applies a random-effects model for data pooling analysis to improve the robustness of the estimated results and ensure consistency in model selection. A funnel plot was applied to evaluate the existence of publication bias. The surface under the cumulative ranking curve (SUCRA) was calculated to rank the probability of interventions. The SUCRA value ranges from 0% to 100%, with higher values indicating that the intervention ranks higher and has a better effect in all comparisons. Given that most studies did not report outcome measures stratified by gender, we did not conduct gender-based subgroup analysis. STATA 15.0 and Review manager 5.3 software was used for conducting the meta-analysis.

## Results

**FIGURE 1:**
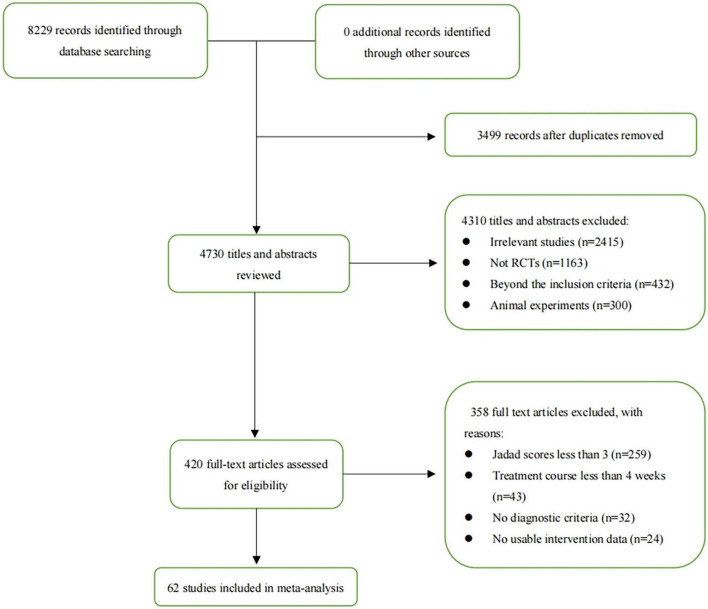
Flowchart of the process for literature retrieval.

The comprehensive literature search identified 8,229 records from the following databases: PubMed, Embase, Springer, Cochrane Controlled Register of Trials (CENTRAL), and Web of Science (the search strategies are provided in Supplementary File 1). After careful screening, 62 studies met the inclusion criteria and were deemed eligible for further quantitative analysis (32–93) is (32–57, 59–95). A flow diagram of the specific screening procedures is shown in Figure 1. The baseline characteristics of the included studies were summarized in Table 1. Totally, 7 interventions were enrolled: FMT, PT, CBT, DI, APMX, standard medical therapy (SMT) such as 5-aminosalicylic acid (5-ASA), and placebo.

**TABLE 1:** Basic characteristic of included studies.

References	Patients				Intervention	Comparison	Duration (weeks)	Outcome	Follow-up (months)	Registration
	Disease type/State of disease	Age (EG/CG)	Gender (M/F)	Country						
Kedia et al. (32)	UC/mild to moderate	37.4/36.9	52/43	India	Coconut water	Placebo	8	Clinical remission; clinical response; endoscopic response; endoscopic remission; Microbiome; FC	N/A	CTRI/2019/03/01827
Narimani et al. (33)	UC/mild to moderate	34.88/39.76	23/25	Iran	Combined Mediterranean, low-FODMAP diet accompanied with partial enteral nutrition	Regular diet	6	SCCAIQ; IBQQ-9; CRP; FC; TAC	N/A	IRCT20100524004010N38
Naude et al. (34)	IBD/mild to moderate	34.3/33.7	22/88	Australia	Acceptance and Commitment Therapy	CBT-Informed psychoeducation program	8	EQ-5D; DASS-21; AAQ-II; BRS; GSE; GUTs; UCAI/CDAI; MIBDI; FSI; PNRS	N/A	ACTRN12621001316897
Haskey et al. (35)	UC/quiescent	47	10/18	Canada	Mediterranean diet pattern	Canadian habitual diet pattern	12	SCCAI; FC; fecal microbial; SCFA	N/A	NCT0305371
Lahtinen et al. (36)	UC/remission	43.0/43.1	26/22	Finland	FMT	Placebo	48	Maintenance of remission; IBDQ; FC	12	NCT03561532
Miyaguchi et al. (37)	UC/remission or mild active	37.5/38	10/10	Japan	Zinc intake and a Japanese diet	Regular diet	24	Clinical remission; UCEIS; CAI; GHS	N/A	UMIN000046664
Bao et al. (38)	CD/mild to moderate	39.5/41.3	41/25	China	Acupuncture plus moxibustion	Sham acupuncture	12	Clinical remission; clinical response; CDAI; CRP; corticosteroid-free remission; CDEIS; recurrence	36	NCT02559037
Chen et al. (39)	UC/mild to moderate	36.7/46.9	6/19	China	Sacral nerve stimulation	sham SNS	8	Mayo scores; clinical response; CRP; FC; Cytokines; Fecal Microbiota Abundance; HRV	N/A	N/A
Goren et al. (40)	CD/moderate to severe	33.6/32.4	41/75	Israel	Cognitive-behavioral and mindfulness-based stress reduction	Wait-list control	12	HBI; GSI; SIBDQ; EQ-5D; FMI; FACIT-F; CRP; FC	N/A	N/A
Haifer et al. (41)	UC/mild to moderate	37.1/36.7	18/17	Australia	FMT	Placebo	8	Clinical remission; clinical response; Endoscopic remission; Endoscopic response; FC; CRP; IBDQ	12	ACTRN 12619000611123
Jedel et al. (42)	UC/inactive	44.8/38.7	21/22	USA	Mindfulness Intervention	Time or attention control	8	UC flare; modified UCDAI; IBDQ; FC; CRP; Mayo Endoscopy Index; Geboe’s score; PSQ; FFMQ; BDI; STAI; PSQI; ECQ	12	NCT01491997
Kedia et al. (43)	UC/mild to moderate	33.9/37.8	40/26	India	FMT	Standard medical therapy	6	Clinical response; clinical remission; Endoscopic response; FC; endoscopic remission	12	ISRCTN15475780
Keshteli et al. (44)	UC/remission	46.5/43	19/34	Canada	Anti-inflammatory diet	Canada’s food guide	24	Clinical relapse; FC; subclinical response rate; gut microbial profiles; SIBDQ	N/A	NCT02093780
Peerani et al. (45)	IBD/stable	45.4/39.7	25/76	Canada	Online stress reduction intervention	Motivational messages by email	12	PSS; partial Mayo scores; HBO; HADS; PSQI; SIBDQ; EQ-5D;PWB; TNF-α; IL-6; IL-10; BDNF; TREM-2; hs-CRP; IDO	6	NCT03831750
Sarbagili Shabat et al. (46)	UC/mild to moderate	43.1/33.3	23/9	Israel	UC exclusion diet	Fecal transplantation	8	Clinical remission; clinical response; SCCAI; endoscopic remission; FC	1	NCT 02734589
Yanai et al. (47)	CD/mild to moderate	26/34	18/22	Israel	Crohn’s disease exclusion diet plus partial nutrition	Crohn’s disease exclusion diet	24	Clinical remission; corticosteroid-free remission; endoscopic remission; FC; CRP	N/A	NCT02231814
Bernabeu et al. (48)	IBD/active	44.5/42	47/73	Spain	Group multicomponent cognitive-behavioral therapy	Treatment as usual	8	PSS; EAE; SRRS; HADS; IBDQ; CDAI; Mayo score	N/A	NCT02614014
Bøezina et al. (49)	UC/mild to moderate	39/39.5	23/22	Prague	FMT	5-ASA	5	Clinical remission; clinical response; endoscopic remission; Mayo score	3	NCT03104036
Crothers et al. (50)	UC/mild to moderate	41/52	7/5	USA	FMT	placebo	12	Clinical remission; clinical response; Mayo scores; IBDQ; CRP; FC; blood T-cells microbiota	9	NCT02390726
Ewais et al. (51)	IBD/NA	22/22	24/41	Australia	Mindfulness-based cognitive therapy	Treatment as usual	8	DASS; Brief COPE; SIBDQ; FFMQ; PTGIl; HCEI; CCKNOW; MAQ; SEAMS; Brief IPQ; ESR; CRP; IL-6; FC; SCCAI; HBI	5	ACTRN12617000876392
Fang et al. (52)	UC/active	51.5/44.6	16/4	China	FMT	Placebo	8	Clinical and mucosa remission; Mayo score; clinical response; clinical symptom scores; fecal microbiota	24	ChiCTR2000030080
Fritsch et al. (53)	UC/remission or mild active	41.7	7/11	USA	A low-fat, high-fiber diet	Improved standard American diet	4	SIBDQ; SF-36; partial Mayo score; FC; CRP; inflammatory markers; microbiome	N/A	NCT04147598
Lacerda et al. (54)	IBD/remission or mild active	44.04/48.32	20/5	Portugal	Mediterranean diet pattern	Regular diet	4	Biochemical evaluation (CRP; FC); Gastrointestinal symptomatology	1	N/A
Lewis et al. (55)	CD/mild to moderate	36/37	70/121	USA	Specific carbohydrate diet	Mediterranean diet	12	Symptomatic remission; FC response; CRP response; SIBDQ; CDAI	N/A	NCT03058679
Cox et al. (56)	IBD/quiescent	33/40	23/29	UK	Low-FODMAP diet	Control diet	4	Symptomatic adequate relief; IBS-SSS; IBDQ; fecal microbiome; FC; CRP	N/A	ISRCTN17061468
González-Moret et al. (57)	IBD/remission	46.2/46.3	19/38	Spain	Mindfulness-based therapy	Standard medical therapy	8	FC; CRP; cortisol in hair	6	N/A
Horta et al. (58)	IBD/quiescent	44.6/38.6/42.6	17/34	Spain	Electroacupuncture	Sham Eac/waitlist	8	FACIT-FS; IBDQ-9; BDI; HAM-A	N/A	NCT02733276
Hunt et al. (59)	IBD/remission or active	35.00/35.69	48/92	USA	Cognitive-behavioral therapy	Psychoeducational workbook	6	HBI; CDAI; GSRS; BSI; GI-COG; SIBDQ; STAI; BDI-II	3	N/A
Jones et al. (60)	CD/stable	46.1/52.3	15/31	UK	Combined impact and resistance training	Without training	6	BMD; muscle function; muscular endurance; IBDQ; EQ-5D; IBD-F; physical activity	6	ISRCTN11470370
Langhorst et al. (61)	UC/remission	50.28/45.54	28/69	German	Comprehensive lifestyle modification	Single two-hour psychoeducational workshop	10	IBDQ; SF-36; CAI; FC; Microbiome; safety	60	NCT02721823
Schierová et al. (62)	UC/active	37.5/40	8/8	Prague	FMT	5-ASA	6	Clinical remission; Clinical response; endoscopic remission; microbial diversity; safety	2	N/A
Seeger et al. (63)	CD/quiescent or mild active	45.3/45.0/43.7	12/22	Germany	Muscle exercise/endurance exercise	Without training	12	CDAI; SIBDQ; SIPAQ; strength increase	6	N/A
Artom et al. (64)	IBD/NA	37/39.13	11/20	UK	Cognitive-behavioral therapy	A short fatigue information sheet to use without therapist help	8	IBD-Fatigue scale; IBDQ; BIPQ; ESS; GAD7 scale; DPHQ9; HBI; SCCAI	12	ISRCTN 17917944
Bodini et al. (65)	IBD/remission or mild	41/47	24/31	Italy	Low-FODMAP diet	Standard diet	6	HBI; Mayo scores; FC; CRP; IBDQ	N/A	N/A
Costello et al. (66)	UC/mild to moderate	38.5/35	40/33	Australia	FMT	Placebo	1	Steroid Clinical remission; clinical response; endoscopic remission; Mayo scores; microbial diversity; adverse events	12	ACTRN12613000236796
Cronin et al. (67)	IBD/quiescent	33/31	15/5	UK	Combined aerobic and resistance training	Without training	8	DEXA; HBI/UCSCI; SF36; HADS; STAI; BDI-II; VO2max; TNF-α; fecal microbiome	N/A	NCT02463916
Sood et al. (68)	UC/active	33/34.6	44/17	India	FMT	Placebo	48	Clinical remission; Endoscopic remission; Histological remission; ESR; CRP	12	CTRI/2018/02/012148
Tew et al. (69)	CD/quiescent or mild active	37.0/38.5/35.0	17/19	UK	High-intensity interval training/moderate-intensity continuous training	Without training	12	CDAI; IBDQ; EQ-5D; HADS; IPAQ; FC	6	ISRCTN13021107
Wynne et al. (70)	IBD/quiescent or stable	40.6/39.9	36/43	Ireland	Acceptance and commitment therapy	Treatment as usual	8	DASS-21; stressometer assessment; AAQ-II; Short Health Scale; short CDAI; short Mayo score; CRP; FC; Hemoglobin; leucocyte; serum albumin concertation; hair cortisol concentration; Hair testosterone and progesterone	3	NCT02350920
Stapersma et al. (71)	IBD/remission or mild	18.62/17.69	22/48	Netherlands	Cognitive-behavioral therapy + standard medical care	Standard medical care	30	SCARED; HADS; BDI-II; CDI; IMPACT-III; IBDQ	N/A	NCT02265588
Bhattacharyya et al. (72)	UC/remission	54/50	8/4	USA	Carrageenan-containing capsules	Placebo	N/A	Clinical relapse; SCCAI; SIBDQ; IL-6,8; TNF-α; NF-κB; B-cell leukemia/lymphoma; FC	12	NCT01065571
Cox et al. (73)	IBD/quiescent	39	11/18	UK	Low-FODMAP diet	Placebo	48	Adequate relief; gastrointestinal symptom incidence; stool frequency and consistency; CRP; FC		ISRCTN98226923
Cramer et al. (74)	UC/remission	45.0/46.1	19/58	Germany	Yoga	Without training	12	IBDQ; UCAI	6	NCT02043600.
Mikocka-Walus et al. (75)	IBD/remission or mild symptoms	N/A	N/A	Australia	Cognitive-behavioral therapy + standard care	Standard care	10	CDAI/SCCAI; CRP; hemoglobin; Platelet; white cell count; SF-36; HADS; STAI; RSRRS; IBD; SCCQ	24	ACTRN12609000913279
Paramsothy et al. (76)	UC/active	35.6/35.4	47/34	Australia	FMT	Placebo	8	Clinical remission; endoscopic remission; Clinical response; endoscopic response; IBDQ; safety	2	NCT01896635
Pedersen et al. (77)	IBD/remission or mild to moderate	22/67	40/41	Denmark	Low-FODMAP diet	Normal diet	6	IBS-SSS; SCCAI; HBI; SIBDQ; response rates; IBS-QOL; FC; CRP	N/A	H-2-2012-05/38987
Bao et al. (78)	CD/remissive	36.61/27.50	24/12	China	Electroacupuncture	Moxibustion	2	CDAI; IBDQ; HRQoL; fMRI scan; ReHo analysis	N/A	NCT01696838
Gunasekeera et al. (79)	CD/consecutive	38/40	32/44	UK	IgG4-Guided exclusion diet	Sham diet	4	SIBDQ; CDAI; HBI; CRP; FC	N/A	N/A
Halmos et al. (80)	CD/quiescent	3/6	35	Australia	Low-FODMAP diet	Typical Australia diet	3	Fecal microbiota; FC; SCFA; gastrointestinal symptom	N/A	N/A
Gerbarg et al. (81)	IBD/mild-to-moderate	49.27/58.57	12/17	USA	Breath-Body-Mind Workshop	Educational seminar	26	BSI-18; BAI; BDI; IBDQ; PDS; PSQ; DDAQ; BIPQ; CRP; FCP	N/A	N/A
Klare et al. (82)	IBD/mild to moderate	39.7/42.5	8/22	Germany	Moderate intensity running	Without training	10	HRQOL; IBDQ; CDAI; CRP; FC; leucocyte; Hemoglobin	N/A	NCT01834573
Moayyedi et al. (83)	UC/active	42.2/35.8	44/31	Canada	FMT	Placebo	6	Clinical remission; improvement in UC symptoms; Mayo score; IBDQ; EQ-5D; CRP; ESR	N/A	NCT01545908
Rossen et al. (84)	UC/mild to moderate	N/A	N/A	Netherlands	FMT	Placebo	3	Clinical remission; Clinical response; endoscopic response; IBDQ; safety	2	NCT01650038
Schoultz et al. (85)	IBD/remission or active	48.59/49.68	10/34	UK	Mindfulness-based cognitive therapy	Wait-list control	8	BDI-II; STAI; MAAS; CDAI/SCCAI; IBDQ	6	ISRCTN27934462
Sharma et al. (86)	IBD/remission	N/A	N/A	India	Yoga + standard medical therapy	Without training	8	HRV; autonomic reactivity; ECP; sIL-2R; STAI	N/A	N/A
Bao (87)	CD/active	36.98/32.38	52/33	China	Herb-partitioned moxibustion + Acupuncture	Herb-partitioned moxibustion + superficial Acupuncture	12	CDAI; hemoglobin; IBDQ; clinical efficacy; CRP; ESR; CDEIS	6	NCT01697761
Berrill et al. (88)	IBD/remission	44.4/45.4	15/51	UK	Muticonvergent therapy + standard medical therapy	Standard medical therapy	16	IBDQ; Clinical relapse; Hassle score; PSQ; IBS-SSS; FC	12	NCT01426568
Jedel et al. (89)	UC/remission	46.04/39.68	24/31	USA	Mindfulness Based Stress Reduction	Time/attention control	8	Disease status; Calprotectin; IL-6, 8, 10; CRP; IBDQ; Cortisol; Fasting Serum ACTH; PSQ; MAAS; STAI; PHCS	12	NCT00568256
Kyaw et al. (90)	UC/NA	N/A	62/50	UK	DMF diet	Usual diet	4 or 6	IBDQ; SCCAI; FFQ	6	N/A
Joos et al. (91)	UC/mild to moderate	39.6/35.8	10/19	Germany	Acupuncture + Moxibustion	Sham acupuncture	5	CAI; IBDQ; VAS; CRP; a1-acid glycoprotein	4	N/A
Ng et al. (92)	CD/remission or mild active	40.6/37.0	14/18	UK	Low intensity exercise	Without training	12	IPAQ; IBDQ; IBDSI; CDAI	N/A	N/A
Joos et al. (93)	CD/active	39.9/36.2	15/36	Germany	Acupuncture + Moxibustion	Control acupuncture	4	CDAI; IBDQ; VAS; CRP	3	N/A

AAQ, action questionnaire; ACTH, adrenocorticotropic hormone; BAI, Beck Anxiety Inventory; BDI, Beck Depression Inventory; BIPQ, brief illness perception questionnaire; BMD, bone mineral density; BSI, brief symptom inventory; BDNF, brain-derived neurotrophic factor; CAI, clinical activity index; CCKNOW, illness knowledge on the Crohn’s and Colitis Knowledge score; CDAI, Crohn’s Disease Activity Index; CDEIS, Crohn’s Disease Endoscopic Index of Severity; CDI, child depression inventory; CG, control group; COPE, coping inventory; CRP, C-reactive protein; DASS, Depression Anxiety and Stress Scales; DDAQ, Digestive Disease Acceptance Questionnaire; DEXA, Dual Energy X-ray Absorptiometry; EAE, perceived stress from the illness; ECP, eosinophilic cationic protein; ECQ, Expectancy and Credibility Questionnaire; EG, experimental group; EQ-5D, EuroQual Five-Dimensional Questionnaire; ESR, erythrocyte sedimentation rate; ESS, Epworth Sleepiness Scales; FACIT, F-Functional Assessment of Chronic Illness Therapy-Fatigue; FC, fecal calprotectin; FSI, Fatigue Symptom Inventory; F, female; FFMQ, five facets of mindfulness questionnaire; FMI, Freiburg mindfulness inventory; GAD7, 7-item Generalized Anxiety Disorder scale; GHS, Geboes Histopathology Score; GI-COG, Gastrointestinal Cognitions Questionnaire; GSI, Global Severity Index; GSRS, Gastrointestinal Symptom Rating Scale; GUTs, Gastrointestinal Unhelpful Thinking Scale; HADS, Hospital Anxiety and Depression Scale; HAM-A, Hamilton Anxiety Rating Scale; HBI, Harvey Bradshaw index; HCEI, Health Care Empowerment Inventory; HRQOL, health-related quality of life; HRV, heart rate variability; IBDQ, Inflammatory Bowel Disease Questionnaire; IBD-SCCQ, IBD Stages of Change Coping Questionnaire; IBDSI, Inflammatory Bowel Disease Stress Index; IBS-SSS, Irritable Bowel Syndrome Symptom Severity Scale; IDO, indoleamine 2,3-dioxygenase; IL, interleukin; IPAQ, International Physical Activity Long Questionnaire; IPQ, Illness Perception Questionnaire; MAAS, State Trait Anxiety Inventory; MAQ, Medication Adherence Questionnaire; MIBDI, Manitoba IBD Index; M, male; PDS, Perceived Disability Scale; PHCS, Perceived Health Competence Scale; PHQ-9, the 9-item Patient Health Questionnaire; PNRS, Pain Numerical Rating Scale; PSQI, Pittsburgh Sleep Quality Index; PSQ, Perceived Stress Questionnaire; PSS, Perceived Stress Scale; PTG, Post-traumatic Growth Inventory; PWB, Psychological Wellbeing Scale; RSRRS, Revised Social Readjustment Rating Scale; SCARED, Screen for Child Anxiety Related Emotional Disorders; SCCAI, Simple Clinical Colitis Activity Index; SCFA, short-chain fatty acid; SEAMS, Self-efficacy on the Appropriate Medication use scale; SF-36, Short Form 36 Health Status Questionnaire; SIBDQ, Short Inflammatory Bowel Disease Questionnaire; sIL-2R, soluble interleukin-2 receptor; SIPAQ, Short International Physical Activity Questionnaire; SRRS, Stressful Life Events Inventory; STAI, State-Trait Anxiety Inventory; TAC, Total Anti-oxidant Capacity; TNF, Tumor Necrosis Factor; UCDAI, Ulcerative Colitis Disease Activity Index; TREM-2, triggering receptor expressed on myeloid cells 2; UCEIS, Ulcerative Colitis Endoscopic Index of Severity; UCSCI, Ulcerative Colitis Simple Colitis Index; VAS, Visual Analog Scale.

### Risk of bias and publication bias

**FIGURE 2:**
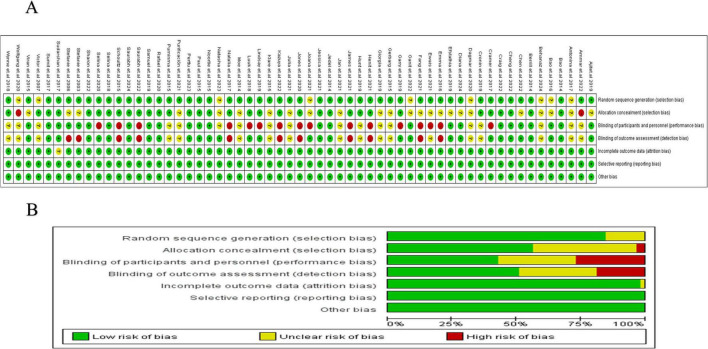
Risk of bias evaluation. **(A)** Risk of bias of each study; **(B)** summary of risk of bias.

**FIGURE 3:**
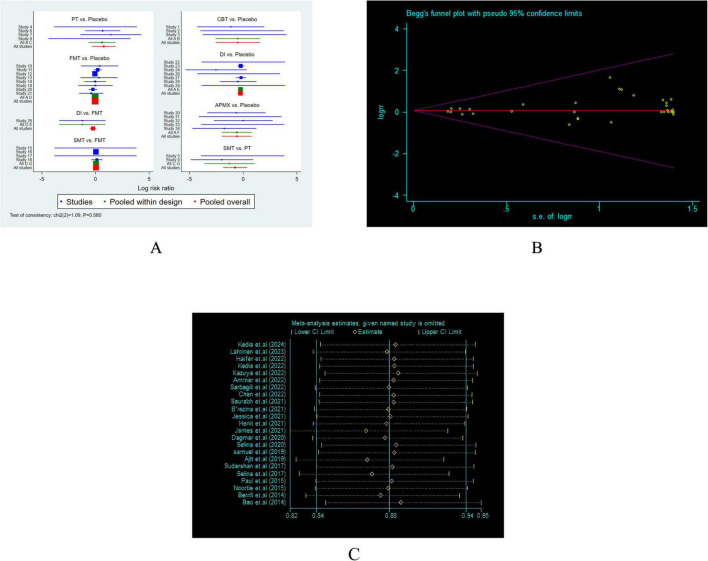
Heterogeneity analysis and Funnel plot. **(A)** Heterogeneity analysis; **(B)** Egger’s regression test. **(C)** Sensitivity analysis.

Among the 62 included studies, 20 were rated as having a high risk of bias in one or more domains, primarily in the areas of blinding of participants and personnel, and blinding of outcome assessment. Additionally, 25 studies were rated as having an unclear risk of bias in two or more domains. A detailed risk of bias assessment is provided in Figure 2. Figure 2A systematically presents the specific assessment of each study across seven risk dimensions, while Figure 2B further summarizes the overall risk distribution for each dimension. Overall, most studies showed low risks in random sequence generation, allocation concealment, and outcome reporting, suggesting a certain level of quality assurance in study design and data reporting. However, some non-pharmacological interventions, due to the limitations of the intervention characteristics (e.g., difficulty in implementing double-blinding), exhibited relatively higher bias risks in blinding-related dimensions, which could impact the internal validity of some outcomes. To further verify the robustness of the results, we conducted a sensitivity analysis. After excluding each study one by one, the changes in the pooled effect size of the main outcomes were minimal, indicating that the study results are stable. The heterogeneity analysis revealed high consistency across studies. The *I*^2^ value was 1.09%, with *P* = 0.580, suggesting strong reliability of the pooled analysis results (Figure 3A). Combined with Egger’s regression test results (*P* > 0.1), no significant publication bias was found (Figure 3B). The primary outcome measures showed minimal variation in the sensitivity analyses, indicating that the overall conclusions of the study remained robust and stable (Figure 3C). Consistency and inconsistency analysis indicate that the data reliability is high, and there is no significant inconsistency or bias in the comparison between treatment methods. In conclusion, the overall bias risk of this study is controllable, heterogeneity is low, and the results demonstrate strong robustness and credibility.

### Clinical remission

#### Induction of remission

**FIGURE 4:**
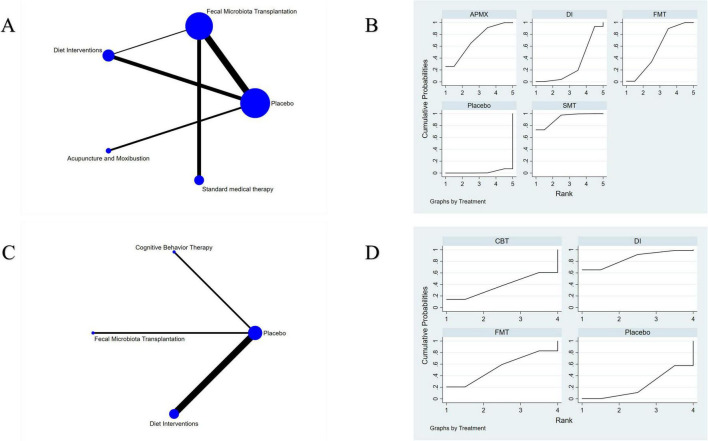
Network meta-analysis of clinical remission. **(A)** network evidence of induction of remission; **(B)** SCURA of induction of remission; **(C)** network evidence of maintenance of remission; **(D)** SCURA of maintenance of remission. The SUCRA results indicate that SMT and DI was the best intervention for the induction and maintenance of clinical remission, respectively.

A total of 19 studies involving 1,120 participants with active IBD were included in the assessment of clinical remission induction (Figure 4A). The results indicated that SMT (RR = 3.02, 95% CI 1.62–5.63), APMX (RR = 2.28, 95% CI 1.23–4.22), and FMT (RR = 1.94, 95% CI 1.22–3.08) therapies were significantly more effective than placebo in inducing clinical remission (Table 2). Additionally, SMT was found to be superior to DI (RR = 2.14, 95% CI 1.05–4.35). The SUCRA plot (Figure 4B) demonstrated that SMT (92.5%) was the most favorable treatment for inducing remission in patients with active IBD, followed by APMX (70.4%) and FMT (56.0%).

**TABLE 2:** Risk ratio or standard mean difference with 95% confidence interval of reported outcomes.

Induction of clinical remission						
**SMT**	**0.75 (0.32, 1.80)**	**0.64 (0.41, 1.00)**	**0.47 (0.23, 0.95)**	**0.33 (0.18, 0.62)**		
**1.33 (0.56, 3.17)**	**APMX**	**0.85 (0.40, 1.83)**	**0.62 (0.29, 1.31)**	**0.44 (0.24, 0.81)**		
**1.56 (1.00, 2.43)**	**1.17 (0.55, 2.51)**	**FMT**	**0.73 (0.42, 1.26)**	**0.52 (0.32, 0.82)**		
**2.14 (1.05, 4.35)**	**1.62 (0.76, 3.41)**	**1.38 (0.79, 2.39)**	**DI**	**0.71 (0.46, 1.10)**		
**3.02 (1.62, 5.63)**	**2.28 (1.23, 4.22)**	**1.94 (1.22, 3.08)**	**1.41 (0.91, 2.19)**	**Placebo**		
**Maintenance of clinical remission**						
**DI**	0.70 (0.27, 1.84)	0.58 (0.19, 1.81)	**0.54 (0.30, 0.97)**			
1.43 (0.54, 3.76)	**FMT**	0.83 (0.24, 2.86)	0.77 (0.36, 1.65)			
1.71 (0.55, 5.33)	1.20 (0.35, 4.10)	**CBT**	0.92 (0.35, 2.42)			
**1.86 (1.03, 3.37)**	1.30 (0.61, 2.78)	1.08 (0.41, 2.85)	**Placebo**			
**Disease activity**						
**APMX**	0.56 (0.24, 1.31)	0.50 (0.20, 1.26)	**0.49 (0.28, 0.88)**	0.46 (0.20, 1.08)	0.40 (0.15, 1.06)	**0.33 (0.17, 0.66)**
1.80 (0.76, 4.24)	**SMT**	0.90 (0.44, 1.84)	0.89 (0.47, 1.67)	0.83 (0.55, 1.25)	0.72 (0.46, 1.13)	0.59 (0.31, 1.12)
2.00 (0.79, 5.03)	1.11 (0.54, 2.27)	**FMT**	0.98 (0.48, 2.02)	0.92 (0.43, 1.99)	0.80 (0.35, 1.87)	0.66 (0.33, 1.34)
**2.03 (1.13, 3.63)**	1.13 (0.60, 2.12)	1.02 (0.49, 2.09)	**Placebo**	0.94 (0.50, 1.75)	0.82 (0.38, 1.77)	**0.67 (0.46, 0.98)**
2.17 (0.92, 5.07)	1.20 (0.80, 1.81)	1.08 (0.50, 2.33)	1.07 (0.57, 1.99)	**CBT**	0.87 (0.48, 1.59)	0.72 (0.37, 1.37)
2.48 (0.94, 6.53)	1.38 (0.88, 2.15)	1.24 (0.54, 2.88)	1.22 (0.56, 2.65)	1.15 (0.63, 2.09)	**PT**	0.82 (0.38, 1.79)
**3.02 (1.51, 6.04)**	1.68 (0.89, 3.18)	1.51 (0.75, 3.07)	**1.49 (1.02, 2.18)**	1.40 (0.73, 2.68)	1.22 (0.56, 2.65)	**DI**
**Quality of life**						
**APMX**	0.95 (0.60, 1.50)	0.81 (0.57, 1.14)	0.76 (0.49, 1.20)	0.74 (0.47, 1.17)	**0.62 (0.47, 0.81)**	
1.05 (0.67, 1.66)	**DI**	0.85 (0.56, 1.30)	0.80 (0.48, 1.34)	0.78 (0.47, 1.31)	**0.65 (0.45, 0.94)**	
1.24 (0.88, 1.74)	1.18 (0.77, 1.80)	**CBT**	0.94 (0.62, 1.43)	0.92 (0.61, 1.39)	0.76 (0.62, 0.94)	
1.31 (0.84, 2.06)	1.25 (0.74, 2.09)	1.06 (0.70, 1.61)	**FMT**	0.97 (0.59, 1.62)	0.81 (0.56, 1.16)	
1.35 (0.86, 2.11)	1.28 (0.76, 2.14)	1.09 (0.72, 1.65)	1.03 (0.62, 1.71)	**PT**	0.83 (0.58, 1.19)	
**1.62 (1.24, 2.13)**	**1.54 (1.07, 2.23)**	1.31 (1.06, 1.62)	1.24 (0.86, 1.78)	1.21 (0.84, 1.73)	**Placebo**	
**Fecal calprotectin**						
**FMT**	0.91 (0.58, 1.43)	0.86 (0.55, 1.34)	0.87 (0.43, 1.77)	0.85 (0.53, 1.35)	0.71 (0.43, 1.16)	
1.09 (0.70, 1.71)	**SMT**	0.94 (0.64, 1.38)	0.95 (0.51, 1.77)	0.93 (0.66, 1.31)	0.78 (0.50, 1.21)	
1.17 (0.75, 1.83)	1.07 (0.73, 1.57)	**Placebo**	1.02 (0.55, 1.86)	0.99 (0.77, 1.27)	0.83 (0.67, 1.02)	
1.15 (0.56, 2.33)	1.05 (0.56, 1.95)	0.98 (0.54, 1.80)	**PT**	0.97 (0.52, 1.82)	0.82 (0.43, 1.55)	
1.18 (0.74, 1.88)	1.08 (0.76, 1.52)	1.01 (0.79, 1.30)	1.03 (0.55, 1.91)	**CBT**	0.84 (0.61, 1.16)	
1.40 (0.86, 2.30)	1.28 (0.83, 1.99)	1.20 (0.98, 1.49)	1.22 (0.64, 2.32)	1.19 (0.86, 1.65)	**DI**	
**C-reactive protein**						
**FMT**	1.08 (0.48, 2.40)	1.15 (0.66, 1.99)	1.23 (0.62, 2.46)	1.27 (0.69, 2.34)	1.33 (0.83, 2.12)	1.55 (0.65, 3.70)
0.93 (0.42, 2.06)	**SMT**	1.07 (0.53, 2.16)	1.14 (0.50, 2.60)	1.18 (0.70, 1.97)	1.23 (0.65, 2.34)	1.44 (0.55, 3.81)
0.87 (0.50, 1.51)	0.94 (0.46, 1.90)	**DI**	1.07 (0.60, 1.91)	1.10 (0.68, 1.79)	1.15 (0.87, 1.53)	1.35 (0.62, 2.95)
0.81 (0.41, 1.62)	0.87 (0.38, 1.99)	0.93 (0.52, 1.67)	**APMX**	1.03 (0.54, 1.95)	1.08 (0.65, 1.79)	1.26 (0.52, 3.06)
0.79 (0.43, 1.45)	0.85 (0.51, 1.42)	0.91 (0.56, 1.47)	0.97 (0.51, 1.84)	**CBT**	1.05 (0.71, 1.54)	1.23 (0.54, 2.80)
0.75 (0.47, 1.21)	0.81 (0.43, 1.55)	0.87 (0.65, 1.15)	0.93 (0.56, 1.54)	0.96 (0.65, 1.41)	**Placebo**	1.17 (0.57, 2.43)
0.64 (0.27, 1.53)	0.69 (0.26, 1.83)	0.74 (0.34, 1.62)	0.79 (0.33, 1.93)	0.82 (0.36, 1.86)	0.85 (0.41, 1.77)	**PT**
**Adverse effect**						
**APMX**	1.40 (0.34, 5.75)	1.10 (0.09, 13.22)	1.76 (0.43, 7.15)	1.84 (0.45, 7.58)	1.82 (0.45, 7.30)	3.91 (0.66, 23.17)
0.72 (0.17, 2.94)	**DI**	0.78 (0.10, 6.28)	1.26 (0.93, 1.70)	1.32 (0.92, 1.88)	**1.30 (1.01, 1.68)**	2.80 (0.90, 8.73)
0.91 (0.08, 11.00)	1.28 (0.16, 10.22)	**CBT**	1.61 (0.20, 12.76)	1.68 (0.21, 13.47)	1.66 (0.21, 13.07)	3.57 (0.34, 37.20)
0.57 (0.14, 2.30)	0.79 (0.59, 1.07)	0.62 (0.08, 4.94)	**FMT**	1.05 (0.87, 1.27)	1.03 (0.88, 1.21)	2.22 (0.73, 6.77)
0.54 (0.13, 2.23)	0.76 (0.53, 1.08)	0.59 (0.07, 4.76)	0.96 (0.79, 1.16)	**SMT**	0.99 (0.77, 1.26)	2.12 (0.69, 6.53)
0.55 (0.14, 2.21)	**0.77 (0.60, 0.99)**	0.60 (0.08, 4.76)	0.97 (0.82, 1.14)	1.01 (0.79, 1.30)	**Placebo**	2.15 (0.71, 6.52)
0.26 (0.04, 1.51)	0.36 (0.11, 1.11)	0.28 (0.03, 2.92)	0.45 (0.15, 1.37)	0.47 (0.15, 1.45)	0.46 (0.15, 1.41)	**PT**

The bold values indicate statistically significant differences (*P* < 0.05). APMX, acupuncture and moxibustion; CBT, cognitive behavior therapy; DI, diet intervention; FMT, fecal microbiota transplantation; PT, physical training; SMT, standard medical therapy.

#### Maintenance of remission

Seven studies involving 365 patients with inactive IBD evaluated the maintenance of remission (Figure 4C). The results suggested that DI was more effective than placebo in maintaining clinical remission (RR = 1.86, 95% CI 1.03–3.37) (Table 2). The SUCRA plot (Figure 4D) indicated that DI ranked highest (85.6%), followed by FMT (61.2%) and CBT (50.3%).

### Consistency analysis and GRADE estimates

**FIGURE 5:**
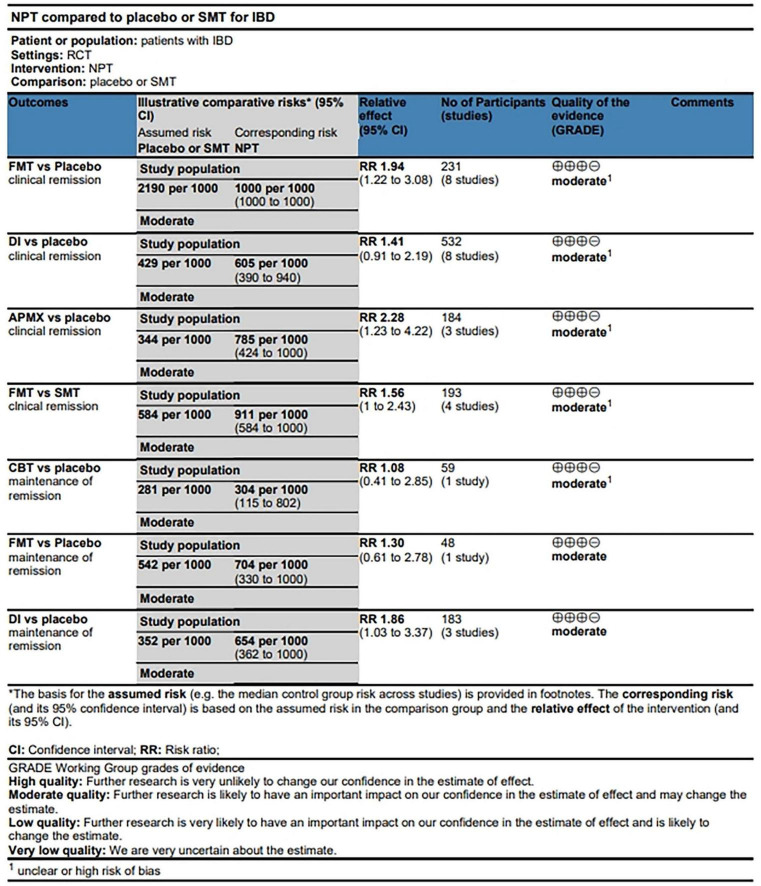
Grading of recommendations assessment, development and evaluation grading assessment.

The results of consistency and inconsistency analysis were shown in Supplementary File 2. The test of consistency showed that chi^2^(1) = 1.78, *P* = 0.182, suggesting good consistency of the model. The node splitting results also showed no inconsistency (all *P* > 0.05). The quality of estimate based on GRADE criteria for clinical remission and maintenance remission (Figure 5) was “moderate,” which was possibly derived from the direct and indirect comparisons within RCTs, leading to imprecision and unclear risk of bias.

### Disease activity

**FIGURE 6:**
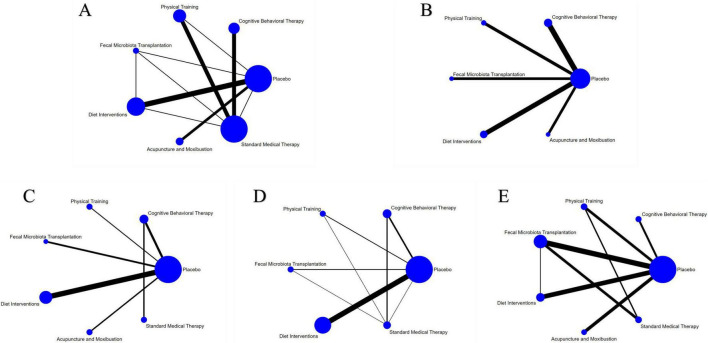
Network meta-analysis of clinical outcomes. **(A)** Disease activity; **(B)** quality of life; **(C)** C-reactive protein; **(D)** fecal calprotectin; **(E)** adverse effects.

**FIGURE 7:**
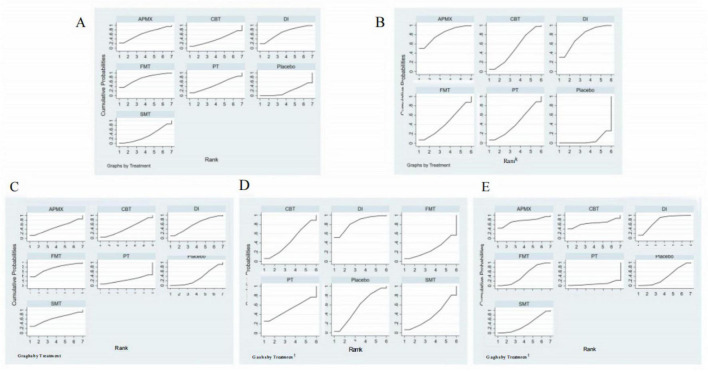
SUCRA of clinical outcomes. **(A)** Disease activity; **(B)** Quality of life; **(C)** C-reactive protein; **(D)** Fecal calprotectin; **(E)** Adverse effects. The SUCRA results indicate that APMX was the best intervention for alleviating disease activity and improving quality of life, while FMT and DI was the most effective treatments for reducing CRP and FC, respectively.

A total of 30 studies involving 1,400 participants assessed disease activity (Figure 6A). Among them, 13 studies used the Crohn’s Disease Activity Index (CDAI), 10 used the Simple Clinical Colitis Activity Index (SCCAI), 3 used the Clinical Activity Index (CAI), and 2 used the UC Disease Activity Index (UCDAI). The network meta-analysis (NMA) results (Table 2) revealed that APMX was superior to placebo (SMD = 2.03, 95% CI 1.13–3.63) and diet interventions (SMD = 3.02, 95% CI 1.51–6.04) in alleviating disease activity. The SUCRA plot (Figure 7A) indicated that APMX (95.9%) was the most effective non-pharmacological treatment for reducing disease activity in IBD, followed by SMT (67.6%) and CBT (52.1%).

### Quality of life

Thirty-seven studies involving 2,312 participants assessed changes in quality of life (Figure 6B). Most studies used the IBD Questionnaire (IBDQ), while 8 studies used the Short IBDQ (SIBDQ), and 2 used the IBDQ-9. The NMA results (Table 2) showed that APMX (SMD = 1.62, 95% CI 1.24–2.13) and DI (SMD = 1.54, 95% CI 1.07–2.23) were more effective than placebo in improving quality of life scores. The SUCRA plot (Figure 7B) suggested that APMX (84.8%) was the most effective non-pharmacological option for improving quality of life in IBD patients, followed by DI (76.0%) and CBT (51.8%). The definition of clinical outcomes were shown in Supplementary File 3.

### Biomarkers of inflammation

C-reactive protein (CRP) was reported in 30 studies with 1,450 patients, while fecal calprotectin (FC) was reported in 26 studies with 1,213 patients (Figures 6C, D). No significant differences in CRP or FC changes were observed between the various treatments (Table 2). However, the SUCRA plots recommended FMT (74.7%) and DI (84.1%) as the most effective treatments for reducing CRP and FC, respectively (Figures 7C, D).

### Adverse effects

Thirty-five studies involving 1,906 participants reported adverse effects (Figure 6E). Compared with placebo, DI was associated with a lower incidence of adverse effects (OR = 0.77, 95% CI 0.60–0.99). No significant differences were found among the non-pharmacological therapies, suggesting that all interventions were similarly safe (Table 2 and Figure 7E).

## Discussion

UC and CD are progressive diseases, and without timely and effective intervention, they can result in irreversible long-term complications (96). Clinical symptoms were once considered a primary factor in evaluating treatment efficacy; however, there is a clear disconnect between clinical symptoms and active mucosal inflammation in IBD, especially in Crohn’s disease (CD) (27, 28). To address this, various diagnostic and monitoring tools have been developed, including clinical symptom-based scoring systems, patient-reported outcomes, serum biomarkers, stool biomarkers, imaging modalities, and ileo-colonoscopy (97).

In this study, we evaluated the following outcomes: clinical remission, disease activity, quality of life (QOL), serum biomarkers (fecal calprotectin [FC] and C-reactive protein [CRP]), and adverse effects. Clinical remission was defined using symptom scoring systems, primarily the Crohn’s Disease Activity Index (CDAI) for CD and the Mayo score for ulcerative colitis (UC) (Supplementary File 2). Disease activity was measured using the Simple Clinical Colitis Activity Index (SCCAI), CDAI, Ulcerative Colitis Disease Activity Index (UCDAI), and Clinical Activity Index (CAI). These scores are simple to implement in clinical practice and are useful for monitoring symptoms over time. In addition to the physical damage caused by IBD, psychosocial issues often arise, with low QOL scores and high disability levels being associated with increased indirect medical costs (98). The most widely used QOL measurement tools in IBD are the IBD Questionnaire (IBDQ) and its shortened version, the Short IBDQ (SIBDQ), both of which were predominantly evaluated in this NMA. Serum biomarkers such as CRP and FC are commonly used non-invasive markers of inflammation in IBD. CRP is generally used to monitor inflammation but has only a moderate correlation with endoscopic disease activity, exhibiting high specificity but low sensitivity for endoscopically active disease (pooled specificity 0.92, 95% CI 0.72–0.96; pooled sensitivity 0.49, 95% CI 0.34–0.64) (99). In contrast, FC has been shown to accurately differentiate between active and quiescent disease in both UC and CD, making it an excellent surrogate marker of mucosal inflammation (99).

The results of NMA show that APMX is the most effective non-pharmacological intervention for inducing clinical remission, alleviating disease activity, and improving patients’ quality of life. Acupuncture and moxibustion are two widely used treatments forms of traditional Chinese medicine and have been employed extensively for the prevention and treatment of IBD, particularly in Asia (38). These treatments are general regarded as natural and safe (100). Acupuncture involves the insertion of slender needles into specific anatomical locations on the body, while Moxibustion applies heat from dried moxa plants to targeted skin areas. Both acupuncture and moxibustion have been reported to alleviate intestinal inflammation, regulate gut microbiota, and relieve IBD symptoms in both animal models and clinical trials (38). In IBD mouse models, APMX has been shown to increase levels of anti-inflammatory cytokines such as interleukin (IL)-10, while decreasing levels of pro-inflammatory cytokines like tumor necrosis factor-alpha (TNF-α), nuclear factor kappa B (NF-κB), IL-6, IL-1β, and IL-17 (101). Electroacupuncture (EA), a form of acupuncture, has been observed to improve colitis severity by maintaining epithelial tight junction proteins and vasoactive intestinal peptide (VIP) receptors, especially VPAC2 (102). Another study found that EA can stimulate neurogenic inflammation, as indicated by reduced levels of substances P, hyaluronic acid, bradykinin, and prostacyclin in the skin of rats with colitis, specifically in the L6 DRG region (103). Clinical studies have demonstrated that APMX can significantly reduce serum TNF-α levels and alter the fecal microbiota composition in UC patients (39). In CD, APMX can enhance the abundance of anti-inflammatory bacteria, improve the intestinal barrier, and regulate circulating Th1/Th17 cytokines (38). In line with the former studies, this NMA show the promising therapeutic effects of APMX in the treatment of IBD, reducing patients’ clinical symptoms and improving their quality of life.

DI were also found to be highly effective, particularly in the maintenance of clinical remission. This aligns with findings from a recent meta-analysis (104). The relationship between diet and IBD is gaining increasing attention, as specific dietary components are recognized for their potential to influence gut health and microbial balance. In this NMA, the most commonly studied dietary interventions included the Mediterranean diet and the low-FODMAP diet. These diets are characterized by low-fat, high-fiber, moderate protein intake (rich in Omega-3 fatty acids), and a reduction in processed foods and sugars (105, 106). The International Organization for the Study of inflammatory bowel disease (IOIBD) has issued guidelines emphasizing the importance of increasing Omega-3 fatty acid intake, particularly from sources like fish oil and fresh fish (107). Omega-3 fatty acids are known for their anti-inflammatory properties and have been shown to alleviate symptoms and promote gut health in UC patients (108). For CD patients, a diet rich in fruits and vegetables, and a reduction in saturated fats, trans fats, dairy fats, additives like polysorbate 80, and artificial sweeteners such as sucralose and saccharin have been recommended (107).

The role of gut microbiota in the development of IBD has recently been emphasized, thus several therapeutic strategies have focused on manipulation of the gut microbiota. Probiotics and antibiotics have been widely used for the treatment of IBD by controlling the growth of pathological organisms, but the results were controversial (109–112). In contrast to both antibiotics and probiotics, FMT may represent a more robust method of manipulating the gut microbiota as a therapy for patients with. Unlike antibiotics, FMT increases the diversity of fecal bacterial populations in recipients, likely contributing to its success in *C. difficile* infection (113, 114). Furthermore, unlike probiotics, evidence suggests that FMT results in long-term engraftment in recipients with *C. difficile* infection (115). Many studies had reported therapeutic effect of FMT in inducing short-term clinical remission/response and changes in disease activity indices, biochemical indicators, and microbial diversity indices for IBD (116). All together, these factors suggest that FMT may be a more promising therapy for IBD than either antibiotics or probiotics. However, donor screening standardization (e.g., microbiome diversity thresholds) route of administration (colonoscopy vs. oral capsules), and regulatory hurdles (e.g., FDA classification as an investigational drug) currently limit widespread adoption.

Although this study found no significant intergroup differences in reducing CRP and FC with NPTs, the SCURA ranking suggests that FMT and DI are the best choices for regulating CRP and FC, respectively. As a systemic inflammation marker, CRP is easily influenced by extrinsic factors (such as infections and stress), and its short half-life (approximately 19 h) may dilute the signal of treatment efficacy (117). Although FC is highly sensitive to local intestinal inflammation, its changes lag behind the improvement of clinical symptoms, and some studies with insufficient follow-up periods (≤ 4 weeks) or inconsistent testing time points may weaken the effect size (118). In addition, FMT inhibits systemic inflammation by transplanting functional microbiota, but its effect on CRP may take longer (≥ 8 weeks) to manifest (76); DI (such as a low FODMAP diet) directly reduces FC release by decreasing intestinal fermentation substrates, and polyphenolic substances (such as oleuropein from olive oil) can quickly inhibit the NF-κB pathway, reducing neutrophil infiltration (56). Overall, the effects of FMT and DI on CRP and FC still need to be further validated in the future.

Another explanation for the lack of significant changes in inflammatory markers (such as CRP) in our study is that we believe these interventions may be more focused on improving symptoms (such as pain and diarrhea) and quality of life, rather than directly reducing baseline inflammation levels. Existing research has shown that the correlation between self-reported symptoms and inflammatory markers is often weak. For example, Gracie et al. (119) noted that the clinical disease activity index does not strongly correlate with endoscopic mucosal inflammation and FCP levels. Seaton et al. (22) also found that psychological factors (such as depression) significantly affect self-reported symptoms, while these symptoms are not closely related to inflammatory markers (22). Similar studies have suggested that, despite no significant changes in inflammatory markers such as FCP, improvements in symptoms and quality of life can still be significant (120–122). Future studies could further explore the mechanisms behind this discrepancy, particularly the role of psychological factors in symptom perception. Such research could help optimize treatment strategies for IBD by integrating these factors.

While our study does incorporate cognitive behavioral therapy (CBT) as part of the psychological intervention, we acknowledge that there may be some differences compared to other studies in the specific application or methodology of CBT. In our study, CBT was combined with other non-pharmacological interventions, such as acupuncture/moxibustion, dietary interventions, and fecal microbiota transplantation. Although these interventions have shown positive results in improving clinical symptoms and disease activity, they may not have had a significant impact on inflammatory markers (such as CRP and FCP) due to differences in the mechanisms of action compared to the interventions described in other studies.

Although we used CBT in our study, there may be differences in terms of the intensity of the intervention, its duration, or the characteristics of the participants when compared to formal study (21). These differences could explain why the impact of CBT on CRP and FCP in our study was not as pronounced as in studies that specifically focused on emotional and psychological health.

Additionally, as noted by Riggott, the effects of psychological interventions can vary significantly depending on study design, participant characteristics, and the intervention methods used (123). While CBT is beneficial in alleviating psychological symptoms such as anxiety and depression, more intensive or longer-term interventions may be necessary to observe significant changes in inflammatory markers. The intervention protocol in our study may have differed in these respects from other studies.

This NMA is, to our knowledge, the first study to compare and summarize the effectiveness and safety of non-pharmacological treatments in IBD patients. It was registered in PROSPERO prior to commencement and followed PRISMA guidelines. All the studies included in this analysis were randomized controlled trials (RCTs), with Jadad scores above 3 points, ensuring a high level of evidence. The outcomes assessed incorporated both clinical manifestations and patient-reported outcomes, combining objective measures with subjective descriptions, allowing for a more comprehensive assessment of treatment efficacy.

However, several limitations must be acknowledged. Firstly, the heterogeneity among the different interventions, variations in treatment courses, indirect comparisons, and differences in outcome measures are notable limitations. While statistical homogeneity was observed across studies, clinical heterogeneity in patient characteristics (e.g., disease duration, baseline severity), treatment protocols (e.g., APMX modalities, FMT donor selection criteria), and outcome definitions (e.g., varying thresholds for clinical remission) may limit the generalizability of our findings. Future research should standardize core outcome measures for NPTs in IBD to enhance cross-trial comparability. Secondly, the sample sizes in many studies were relatively small, and nearly half of the studies were assessed as having an “unclear or high risk of bias” in terms of selection, performance, and detection biases. Thirdly, this study mainly explores the short-term to mid-term efficacy of NPTs, so the long-term effects of NPTs needed further investigation. Additionally, a key limitation of our study is the broad categorization of interventions. Future research should refine the intervention types (such as different dietary patterns, psychological therapies, and exercise intensities) to assess the specific efficacy of each intervention on IBD. This approach will not only help to reveal the differences in the effects of various interventions but will also provide more targeted guidance for non-pharmacological treatments of IBD. Besides, One of the main limitations of this network meta-analysis is the heterogeneity in the reporting and definitions of adverse effects and biomarker outcomes across the included studies. Different studies used varying definitions of adverse events and biomarker measurements, making it difficult to directly compare results across studies. Additionally, some studies did not report certain adverse effects or biomarkers, further complicating our analysis. Due to insufficient sample size, a detailed subgroup analysis could not be performed. The absence of subgroup analysis may limit our assessment of the applicability and external validity of the treatment effects across different patient populations. Despite these limitations, our study provides valuable insights into the potential benefits of non-pharmacological interventions for IBD. SUCRA ranking provides only a relative ranking of treatments. In clinical practice, it should be considered alongside the quality of evidence for each outcome, the risk of bias, and the clinical significance of the treatment effects for a comprehensive evaluation. By addressing these limitations in future research, we can better understand the nuances of each intervention and improve the precision of treatment recommendations, ultimately enhancing patient care and outcomes in IBD management.

## Conclusion

This NMA provides comprehensive insights into the efficacy and safety of common non-pharmacological interventions for IBD. The results highlight that APMX is the most effective treatment for inducing clinical remission, alleviating disease activity, and improving quality of life. DI are best for maintaining clinical remission and reducing serum FC levels, while FMT was found to be the most effective at reducing serum CRP levels. These findings underscore the promising potential of non-pharmacological treatments in managing IBD and improving patient outcomes. Although APMX shows potential as a non-pharmacological intervention for inflammatory bowel disease, the current evidence is limited by methodological shortcomings and heterogeneity among studies. Therefore, further high-quality, rigorously designed randomized controlled trials are warranted to confirm its efficacy and safety. In the meantime, the current evidence should be applied cautiously in clinical decision-making. While caution is warranted in the clinical application of APMX due to the current limitations in evidence quality, it is encouraging that several other non-pharmacological interventions demonstrated promising efficacy. These findings offer patients a broader range of scientifically supported treatment options, laying the groundwork for personalized care and empowering individuals to make informed decisions based on their unique needs and preferences.

### Strength and limitations

1. To our knowledge, this is the first network meta-analysis comparing the effectiveness and safety of non-pharmacological treatment for IBD. The study evaluated a wide range of outcomes, including clinical remission, disease activity, quality of life (QOL), serum biomarkers (FC and CRP), and adverse effects, offering a holistic view of treatment efficacy and safety.

2. The use of network meta-analysis (NMA) allowed for direct and indirect comparisons of multiple NPTs, providing a robust ranking of interventions (e.g., APMX, DI, FMT) based on their effectiveness across diverse outcomes.

3. The application of Cochrane Handbook guidelines and GRADEpro software for quality assessment, along with funnel plots to evaluate publication bias, enhanced the reliability and transparency of the findings. This study emphasizes the management of mental health issues in IBD patients, evaluating the effectiveness of psychological interventions like cognitive behavioral therapy, reflecting the growing importance of comprehensive care for IBD patients, and aligning with the current trends in IBD management.

4. Variability in study designs, patient populations, and intervention protocols across the included RCTs may have introduced heterogeneity, potentially affecting the consistency and generalizability of the findings. Despite the use of funnel plots, the possibility of unpublished negative results or selective reporting in the included studies could not be entirely ruled out, potentially skewing the findings.

5. The reliance on aggregated data from published RCTs limited the ability to perform patient-level analyses or adjust for confounding factors that may have varied across studies.

## Data Availability

The original contributions presented in this study are included in this article/Supplementary material, further inquiries can be directed to the corresponding authors.

## Supplementary material

Supplementary File 1 Search strides.

Supplementary File 2 Consistency and inconsistency analysis.

Supplementary File 3 Definition of clinical outcomes.
