# Supplementary material for: Effectiveness and safety of non-pharmacological therapies for the treatment of inflammatory bowel disease: a network meta-analysis
Source: Front Med (Lausanne). 2025 Jun 30;12:1593483. doi: 10.3389/fmed.2025.1593483 (PMC12256550; doi:10.3389/fmed.2025.1593483)
Supplement: Supplementary File 1 — Search strides. [file Data_Sheet_1.pdf]

Search strategy

Pubmed

(("inflammatory bowel disease"[MeSH] OR "crohn disease"[MeSH] OR "colitis, ulcerative"[MeSH] OR "IBD"[tiab] OR "UC"[tiab] OR "CD"[tiab] OR "inflammatory bowel*"[tiab] OR "ulcerative colitis"[tiab] OR "crohn*"[tiab])

AND (("cognitive behavioral therapy"[MeSH] OR "mind-body therapies"[MeSH] OR "mindfulness"[MeSH] OR "CBT"[tiab] OR "MBI"[tiab] OR "MT"[tiab] OR "mind body therap*"[tiab] OR "multi-convergent therap*"[tiab] OR "mindfulness-based"[tiab])

OR ("diet therapy"[MeSH] OR "nutrition therapy"[MeSH] OR "diet* intervention*"[tiab] OR "nutrition* therap*"[tiab] OR "dietary treatment*"[tiab] OR "food intervention*"[tiab])

OR ("fecal microbiota transplantation"[MeSH] OR "FMT"[tiab] OR "intestinal microbiota transplant*"[tiab] OR "bacteriotherap*"[tiab])

OR ("exercise"[MeSH] OR "exercise therapy"[MeSH] OR "physical activit*"[tiab] OR "physical train*"[tiab] OR "resistance train*"[tiab] OR "endurance train*"[tiab] OR "yoga"[tiab])

OR ("acupuncture therapy"[MeSH] OR "moxibustion"[MeSH] OR "electroacupuncture"[MeSH] OR "acupuncture"[tiab] OR "moxibustion"[tiab] OR "electro-acupuncture"[tiab]))

AND (“randomized controlled trial"[Publication Type] OR "controlled clinical trial"[Publication Type] OR "RCT"[tiab] OR "random*"[tiab]))

NOT (animal[mh] NOT human[mh])

Embase

(('inflammatory bowel disease'/exp OR 'crohn disease'/exp OR 'ulcerative colitis'/exp OR ibd:ti,ab OR uc:ti,ab OR cd:ti,ab OR 'inflammatory bowel*':ti,ab OR 'ulcerative colitis':ti,ab OR Crohn*: ti,ab)

AND (('cognitive behavioral therapy'/exp OR 'mind body therapy'/exp OR 'mindfulness'/exp OR cbt:ti,ab OR mbi:ti,ab OR mt:ti,ab OR 'mind body therap*':ti,ab OR 'multi-convergent therap*':ti,ab OR 'mindfulness-based':ti,ab)

OR ('diet therapy'/exp OR 'nutrition'/exp OR 'diet* intervention*':ti,ab OR 'nutrition* therap*':ti,ab OR 'dietary treatment*':ti,ab OR 'food intervention*':ti,ab)

OR ('fecal microbiota transplantation'/exp OR fmt:ti,ab OR 'intestinal microbiota transplant*':ti,ab OR bacteriotherap*:ti,ab)

OR ('exercise'/exp OR 'physical activity'/exp OR 'physical activit*':ti,ab OR 'physical

train*':ti,ab OR 'resistance train*':ti,ab OR 'endurance train*':ti,ab OR yoga:ti,ab)

OR ('acupuncture'/exp OR 'moxibustion'/exp OR 'electroacupuncture'/exp OR acupuncture:ti,ab OR moxibustion:ti,ab OR electro-acupuncture:ti,ab))

AND (‘randomized controlled trial'/exp OR 'controlled clinical trial'/exp OR rct:ti,ab OR random*:ti,ab))

NOT ([animals]/lim NOT [humans]/lim)

Spring

((TITLE:(("inflammatory bowel disease" OR Crohn* OR "ulcerative colitis" OR IBD OR UC OR CD)) OR ABSTRACT:(("inflammatory bowel disease" OR Crohn* OR "ulcerative colitis" OR IBD OR UC OR CD)))

AND ((TITLE:("cognitive behavioral therapy" OR CBT OR "mind body therapy*" OR mindfulness OR MBI OR MT OR "multi-convergent therapy*") OR ABSTRACT:("cognitive behavioral therapy" OR CBT OR "mind body therap*" OR mindfulness OR MBI OR MT OR "multi-convergent therapy*"))

OR (TITLE:("diet intervention*" OR "diet therapy*" OR nutrition* OR "dietary treatment*" OR "food intervention*") OR ABSTRACT:("diet intervention*" OR "diet therapy*" OR nutrition* OR "dietary treatment*" OR "food intervention*"))

OR (TITLE:("fecal microbiota transplantation" OR FMT OR "intestinal microbiota transplant*" OR bacteriotherapy*) OR ABSTRACT:("fecal microbiota transplantation" OR FMT OR "intestinal microbiota transplant*" OR bacteriotherapy*))

OR (TITLE:("physical activity*" OR "physical train*" OR exercise* OR "resistance train*" OR "endurance train*" OR yoga) OR ABSTRACT:("physical activity*" OR "physical train*" OR exercise* OR "resistance train*" OR "endurance train*" OR yoga))

OR (TITLE:(acupuncture OR moxibustion OR electro-acupuncture) OR ABSTRACT:(acupuncture OR moxibustion OR electro-acupuncture)))

AND (TITLE:("randomized controlled trial" OR RCT OR random* OR "controlled clinical trial") OR ABSTRACT:("randomized controlled trial" OR RCT OR random* OR "controlled clinical trial"))

Cochrane Controlled Register of Trials (CENTRAL)

((inflammatory bowel disease OR crohn* OR "ulcerative colitis" OR IBD OR UC OR CD):ti,ab,kw

AND (("cognitive behavioral therapy" OR CBT OR "mind body therap*" OR mindfulness OR MBI OR MT OR "multi-convergent therap*")

OR ("diet intervention*" OR "diet therap*" OR nutrition* OR "dietary treatment*"

OR "food intervention*")

OR ("fecal microbiota transplantation" OR FMT OR "intestinal microbiota transplant*" OR bacteriotherap*)

OR ("physical activit*" OR "physical train*" OR exercise* OR "resistance train*" OR "endurance train*" OR yoga)

OR (acupuncture OR moxibustion OR electro-acupuncture))

AND (“randomized controlled trial" OR RCT OR random* OR "controlled clinical trial"))

NOT (animal* NOT human*)

Web of Science

(TS= (("inflammatory bowel disease" OR Crohn* OR "ulcerative colitis" OR IBD OR UC OR CD)

AND (("cognitive behavioral therapy" OR CBT OR "mind body therapy*" OR mindfulness OR MBI OR MT OR "multi-convergent therapy*")

OR ("diet intervention*" OR "diet therapy*" OR nutrition* OR "dietary treatment*" OR "food intervention*")

OR ("fecal microbiota transplantation" OR FMT OR "intestinal microbiota transplant*" OR bacteriotherapy*)

OR ("physical activity*" OR "physical train*" OR exercise* OR "resistance train*" OR "endurance train*" OR yoga)

OR (acupuncture OR moxibustion OR electro-acupuncture))

AND (“randomized controlled trial" OR RCT OR random* OR "controlled clinical trial”)) AND DT= (Article OR Clinical Trial))

NOT TS= (animal* NOT human*)
