# Supplementary material for: Effectiveness and safety of non-pharmacological therapies for the treatment of inflammatory bowel disease: a network meta-analysis
Source: Front Med (Lausanne). 2025 Jun 30;12:1593483. doi: 10.3389/fmed.2025.1593483 (PMC12256550; doi:10.3389/fmed.2025.1593483)
Supplement: Supplementary File 2 — Consistency and inconsistency analysis. [file Data_Sheet_2.pdf]

Supplementary 2. Definition of clinical outcomes

study	Clinical remission	Disease activity	Quality of life
Behanaz et.al 2024	NA	SCCAI	IBDQ-9
Diana et.al 2024	NA	NA	IBDQ-9
Naude et.al 2024	NA	UCAI/CDAI	NA
Sajjad et.al 2024	NA	SCCAI	NA
Saurabh et.al 2024	SCCAI≤2	NA	NA
Natasha et.al 2023	NA	SCCAI	NA
Perttu et.al 2023	Mayo score ≤ 2, FC<200 μg/g	NA	IBDQ
Perttu et.al 2023	NA	NA	NA
Ammar et.al 2022	FCP < 150 µg/g	NA	NA
Bao et.al 2022	CDAI score <150 and decrease ≥70 from baseline	CDAI	NA
Chen et.al 2022	SCCAI≤2	SCCAI	NA
Chen et.al 2022	reduced Mayo score≥2, at least 25% (compared with pretherapy) level) with an accompanying decrease in the rectal bleeding Mayo subscore of ≥ 1 point or an absolute rectal bleeding Mayo subscore of 0 to 1	NA	NA
Craig et.al 2022	Mayo score ≤2, all subscore≤1, ≥1 point reduction in endoscopic subscore endoscopy subscore	NA	NA
Ganit et.al 2022	NA	NA	SIBDQ
Kazuya et.al 2022	CAI≤4	CAI	NA
Peerani et.al 2022	NA	NA	SIBDQ
Saurabh et.al 2022	SCCAI<2, endoscopic UCEIS <1	NA	NA
Sharon et.al 2022	NA	UCDAI	IBDQ

Bˇrezina et.al 2021	Mayo score ≤ 2, all subscore≤1	NA	NA
Ewais et.al 2021	NA	NA	SIBDQ
Henit et.al 2021	HBI<5	CDAI	NA
James et.al 2021	SCDAI≤150	NA	SIBDQ
James et.al 2021	NA	NA	NA
Jessica et.al 2021	Mayo score ≤ 2, all subscore≤1	SCCAI	IBDQ
Jessica et.al 2021	NA	NA	NA
Jessica et.al 2021	NA	NA	NA
Julia et.al 2021	NA	NA	SIBDQ
Purificación et.al 2021	NA	CDAI	IBDQ
Saurabh et.al 2021	SCCAI<2, endoscopic UCEIS <1	SCCAI	NA
Dagmar et.al 2020	Mayo score ≤ 2, all subscore≤1	NA	NA
Jones et.al 2020	NA	NA	IBDQ
Langhorst et.al 2020	NA	CAI	IBDQ
Selina et.al 2020	global symptom question	NA	NA
Wolfgang et.al 2020	NA	CDAI	SIBDQ
Ajit et.al 2019	Mayo score ≤ 2, all subscore≤1	NA	NA
Artom et.al 2019	NA	SCCAI	IBDQ
Cronin et.al 2019	NA	UCSCI	NA
Garry et.al 2019	NA	CDAI	IBDQ
Giorgia et.al 2019	NA	NA	IBDQ
Hunt et.al 2019	NA	NA	SIBDQ

samuel et.al 2019	Mayo score≤2, endoscopic Mayo score ≤1	NA	NA
Luuk et.al 2018	NA	NA	IBDQ
Antonina et.al 2017	NA	CDAI	NA
Cramer et.al 2017	NA	CAI	IBDQ
Natalia et.al 2017	NA	SSCAI	SIBDQ
Selina et.al 2017	global symptom question	NA	NA
Sudarshan et.al 2017	Mayo score ≤2, all subscore≤1, ≥1 point reduction in endoscopic subscore	NA	IBDQ
Sumit et.al 2017	NA	SCCAI	SIBDQ
Sumit et.al 2017	NA	NA	NA
Bao et.al 2016	NA	CDAI	IBDQ
Gerbarg et.al 2015	NA	NA	IBDQ
Klare et.al 2015	NA	CDAI	IBDQ
Noortie et.al 2015	SCCAI score of ≤2, ≥1 point improvement on Mayo endoscopic score	NA	IBDQ
Paul et.al 2015	Mayo scores ≤2, endoscopic Mayo score = 0	NA	IBDQ
Paul et.al 2015	NA	NA	NA
Schoultz et.al 2015	NA	CDAI	IBDQ
Viran et.al 2015	NA	CDAI	SIBDQ
Bao et.al 2014	CDAI≤150	CDAI	IBDQ
Berrill et.al 2014	SCCAI<3	NA	IBDQ
Jedel et.al 2014	NA	UCDAI	IBDQ
Jedel et.al 2014	NA	NA	NA
Moe et.al 2014	NA	SCCAI	BIDQ

Moe et.al 2014	NA	NA	NA
Victor et.al 2007	NA	CDAI	IBDQ
Stefanie et.al 2006	NA	CAI	IBDQ
Stefanie et.al 2004	NA	CDAI	IBDQ

Annotations: NA-not applicable; CAI-Clinical Activity Index; CDAI-Crohn's Disease Activity Index; CDEIS-Crohn’s Disease Endoscopic Index of Severity; FC-fecal calprotectin; HBI-Harvey Bradshaw Index; IBDQ-Inflammatory Bowel Disease Questionnaire; SCCAI-Simple Clinical Colitis Activity Index; SIBDQ-Short Inflammatory Bowel Disease Questionnaire; UCDAI-Ulcerative Colitis Disease Activity Index; UCEIS-Ulcerative Colitis Endoscopic Index of Severity.
