# Supplementary material for: Effectiveness and safety of non-pharmacological therapies for the treatment of inflammatory bowel disease: a network meta-analysis
Source: Front Med (Lausanne). 2025 Jun 30;12:1593483. doi: 10.3389/fmed.2025.1593483 (PMC12256550; doi:10.3389/fmed.2025.1593483)
Supplement: Supplementary File 3 — Definition of clinical outcomes. [file Supplementary_file_1.pdf]

Consistency analysis:

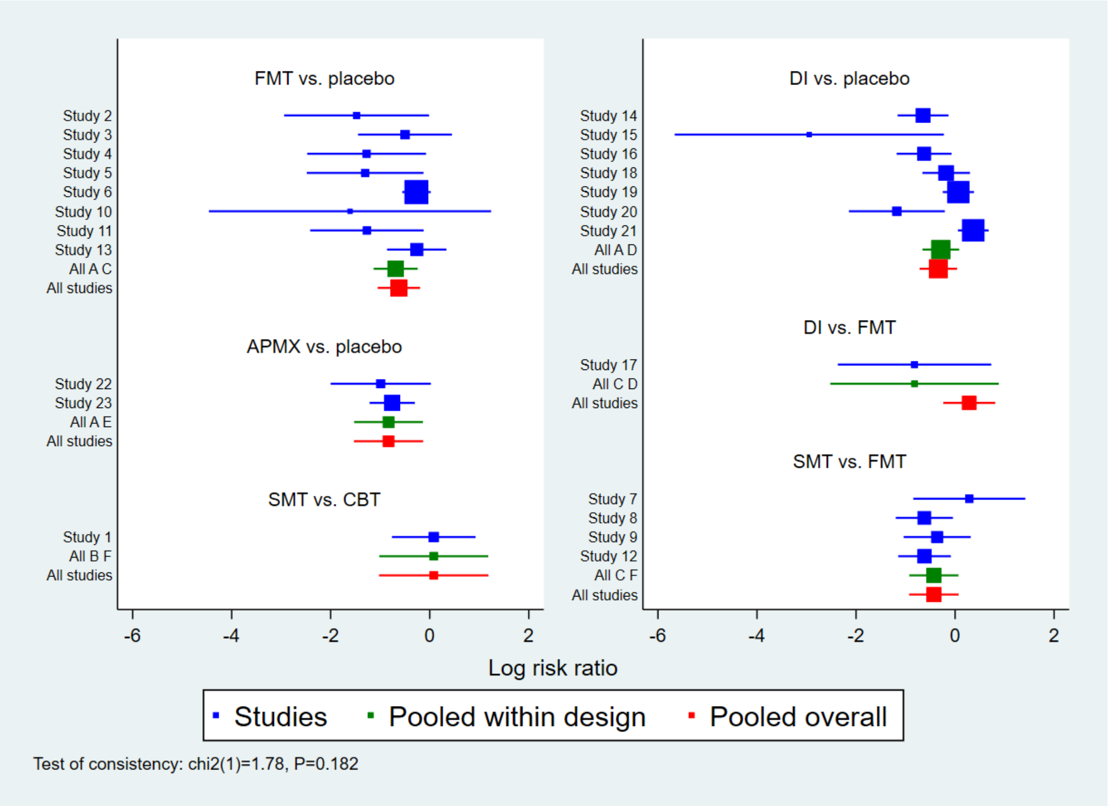

Inconsistency analysis:

Treatment	Direct		Indirect		Difference			tau
	Coef.	Std. Err.	Coef.	Std. Err.	Coef.	Std. Err.	P>|z|	
**CBT vs DI**	0.680254	0.224139	1.127919	0.88304	-0.44766	0.906043	0.621	0.348164
**CBT vs APMX**	0.309638	0.184549	-0.13806	0.892171	0.447694	0.906057	0.621	0.348166
**CBT vs SMT**	.	.	.	.	.	.	.	.
**FMT vs placebo**	0.081415	0.550946	0.54729	1030.278	-0.46587	1030.278	1	0.343307
**DI vs APMX**	-0.81831	0.863556	-0.37062	0.274245	-0.44769	0.906057	0.621	0.348165
**DI vs placebo**	-0.43138	0.24698	-1.20811	287.7038	0.776728	287.7039	0.998	0.343307
